# Increasing Complexity of Ribosomes and Their Biogenesis

**DOI:** 10.3390/ijms23158264

**Published:** 2022-07-27

**Authors:** Lasse Lindahl

**Affiliations:** Department of Biological Sciences, University of Maryland, Baltimore County, 1000 Hilltop Circle, Baltimore, MD 21250, USA; lindahl@umbc.edu

## 1. A Very Short Introduction to Ribosomes and their Biosynthesis

According to the classic ribosome model, developed in the 1960s and 1970s, its only function is to translate the four-letter nucleic acid code into the 20 amino acid peptide-code, while polymerizing amino acids into peptides with the help of a large complement of tRNAs and translation factors that cycle on and off the ribosome [1,2]. However, advances accumulating over the recent decades have shown that the ribosome performs tasks beyond the classic model, such as initial folding of nascent peptides and regulation of translation in response to growth and stress conditions [3,4,5]. Moreover, the ribosome interacts with the Signal Recognition Particle to secure the post-translation transport of protein products to their proper cellular location [6]. 

Given the complexity of the ribosome function, it is not surprising that the ribosome structure has many components of both rRNA and ribosomal proteins (r-proteins) (Table 1). Some r-proteins are phylogenetically preserved and contribute to a universal ribosomal core structure, while others differ between phylogenetic domains [7]. 

Ribosome assembly requires synthesis and modification of its components, which occurs simultaneously with their assembly into ribosomal particles. The pathways differ phylogenetically, but universally the formation occurs by a stepwise ordered addition of ribosome components. The process is assisted by many assembly factors that facilitate and monitor the individual steps, for example by modifying ribosomal components, releasing assembly factors from an assembly intermediate, or forcing specific structural configurations [8,9,10,11,12,13,14]. The quality of the ribosome population is controlled by a complement of nucleases that degrade assembly intermediates with an inappropriate structure and/or which constitute kinetic traps [15]. Ribosomal variants can be formed by alternate pathways or incorporation of paralogous r-protein variants, but the functional significance is generally still not clear [16,17]. Adding to the complexity are the extra-ribosomal functions of some r-proteins as regulators of cell cycle progression and apoptosis, or as substrates for post-translational ubiquitination of protein products [18].

## 2. The Volume

The intent of this special volume is to illustrate selected principles in ribosome biogenesis. Some chapters also address the consequences of impeding ribosome biogenesis, often termed nucleolar stress. For comprehensive reviews of ribosome biogenesis, the reader is referred to other sources (see above). 

Ribosome formation begins in the nucleolus, a membrane-less distinct domain of the nucleus. The morphology of this organelle has been extensively explored in metazoan cells, but the analysis of the yeast nucleolus is challenged by the low contrast of its mostly decondensed chromatin. Thelen, Defourny, Lafontaine, and Thiry show that this hurdle can be overcome by subjecting cells to hyperosmotic shock [19]. Moreover, they find that the organization of the yeast nucleolus into separate domains is less distinct than is the case in mammalian nucleoli.

Two papers illustrate that combination of mutant analysis and cryo-electron microscopy is an effective method for elucidating the function of assembly factors. Paternoga, Früh, Kunze, Bradatsch, Baßler, and Hurt show that the N-terminal domain of the Nsa2 assembly factor binds to the 60S precursor particle, thereby preventing the cytoplasmic release and nuclear reimport of the Nog1 GTPase [20]. Maksimova, Korepanov, Kravchenko, Baymukhametov, Myasnikov, Vassilenko, Afonina, and Stolboushkina classified the bacterial 30S ribosomal subunit according to structure in response to depleting the assembly factor RbfA. The results indicate that RpfA stabilizes the 30S pseudoknot (a universal feature of the small ribosomal subunit across the evolutionary spectrum) and assists the docking of 16S rRNA Helix 44 into the decoding center [21].

Three of the four rRNA components in eukaryotic ribosomes are generated by nucleolytic processing of the RNA PolI primary transcript. The ribozyme RNase MRP is necessary for rRNA processing and ribosome production [22,23,24,25,26]. It cleaves a specific site in the precursor rRNA [27], but the paper by Li, Zengel, and Lindahl now shows that this cleavage is dispensable for rRNA maturation. Based on this and their previously published data, the authors suggest that RNase MRP may be necessary for the activation of other rRNA processing activities [28].

Kofler, Prattes, and Bergler demonstrate that the addition of small molecular-weight inhibitors can be used to interrogate the kinetics of the individual steps in the ribosome formation. Their analysis further shows how the perturbation of individual steps affects the kinetics of other parts of the pathway, both downstream and upstream of the perturbation site [29].

The synthesis of a protein must be followed by transport to its site of function. The Signal Recognition Particle (SRP), anther RNA-protein complex, receives numerous nascent proteins as they emerge from the ribosome exit tunnel and chaperones them to their cellular location. Kellogg, Miller, Tikhonova, and Karamyshev present a review of this process and discuss the composition and evolution, as well as the signals built into individual proteins, which assure their delivery to their appropriate location [30].

Two r-proteins, e-S31 and eL40, are synthesized as fusions with ubiquitin (Ub) entities and are used as substrates for post translational ubiquitination. Martín-Villanueva, Gutiérrez, Kressler, and de la Cruz review the distribution of Ub-encoding genes across the evolutionary spectrum, discuss the various consequences of ubiquitination, and analyze the potential evolutionary pressures that may have contributed to the current state of ubiquitination [31].

Pecoraro, Pagano, Russo, and Russo briefly summarize the major phases of ribosome biogenesis and continue into a review of how r-proteins promote tumorigenesis. Boosted synthesis of either cytoplasmic and mitochondrial r-proteins can cause r-proteins to assume extra-ribosomal functions, including inhibiting turnover of the tumor suppressor p53 or otherwise interfering with cell cycle progression [32].

Mutations in genes that decrease normal ribosome biogenesis or function can lead to numerous congenital diseases, collectively called ribosomopathies [33,34]. The analysis of these diseases is complicated by fact that ribosome deficiencies are the systemic. The paper by DeLeo, Baral, Houser, James, Sewell, Pandey, and DiMario reviews how this hurdle can be overcome by using the imaginal discs of Drosophila [35]. Furthermore, they show inhibition of the synthesis of Nopp140, a protein found in both nucleoli and Cajal bodies (sites for the assembly of several RNA protein particles, including some that help modify rRNA) in several imaginal discs. The ribosome content is reduced, simultaneously with the accumulation of unusual cytoplasmic bodies, sharing several proteins with (P) processing bodies. 

The ribosome formation is somewhat flexible and contributes to the adaptation of organisms to changing environments. Martinez-Seidel, Beine-Golovchuk, Hsieh, Eshraky, Gorka, Cheong, Jimenez-Posada, Walther, Skirycz, Roessner, Kopka, and Firmino report on an extensive analysis of r-protein gene expression in Arabidopsis following a temperature down-shift. Interestingly, changes in the expression of paralogous genes for a given protein depend on the location of the proteins in the ribosome [36]. This suggests that limited flexibility in the ribosome structure may contribute to temperature adaptation.

## Figures and Tables

**Table 1:** Ribosome composition.

Type of Ribosome	Number of rRNAs	Number of Ribosomal Proteins
*Escherichia coli*	3	57
Archaea	3	Up to 68
*Saccharomyces cerevisiae* cytoplasmic	4	79
*Saccharomyces cerevisiae* mitochondrial	2	72
Human	4	80

## References

[1] Lipmann F. (1969). Polypeptide chain elongation in protein biosynthesis. Science.

[2] Anderson W.F., Bosch L., Gros F., Grunberg-Manago M., Ochoa S., Rich A., Staehelin T. (1974). Initiation of protein synthesis in prokaryotic and eukaryotic systems. Summary of EMBO Workshop. FEBS Lett..

[3] Kramer G., Shiber A., Bukau B. (2019). Mechanisms of Cotranslational Maturation of Newly Synthesized Proteins. Annu. Rev. Biochem..

[4] Farache D., Antine S.P., Lee A.S.Y. Moonlighting translation factors: Multifunctionality drives diverse gene regulation. Trends Cell Biol..

[5] Hinnebusch A.G. (2017). Structural Insights into the Mechanism of Scanning and Start Codon Recognition in Eukaryotic Translation Initiation. Trends Biochem. Sci..

[6] Mercier E., Wang X., Bogeholz L.A.K., Wintermeyer W., Rodnina M.V. (2022). Cotranslational Biogenesis of Membrane Proteins in Bacteria. Front. Mol. Biosci..

[7] Ban N., Beckmann R., Cate J.H., Dinman J.D., Dragon F., Ellis S.R., Lafontaine D.L., Lindahl L., Liljas A., Lipton J.M. (2014). A new system for naming ribosomal proteins. Curr. Opin. Struct. Biol..

[8] Naganathan A., Culver G.M. (2022). Interdependency and Redundancy Add Complexity and Resilience to Biogenesis of Bacterial Ribosomes. Annu. Rev. Microbiol..

[9] Mitterer V., Pertschy B. (2022). RNA folding and functions of RNA helicases in ribosome biogenesis. RNA Biol..

[10] Vanden Broeck A., Klinge S. (2022). An emerging mechanism for the maturation of the Small Subunit Processome. Curr. Opin. Struct. Biol..

[11] Klinge S., Woolford J.L. (2019). Ribosome assembly coming into focus. Nat. Rev. Mol. Cell Biol..

[12] Bohnsack K.E., Bohnsack M.T. (2019). Uncovering the assembly pathway of human ribosomes and its emerging links to disease. EMBO J..

[13] Hilander T., Jackson C.B., Robciuc M., Bashir T., Zhao H. (2021). The roles of assembly factors in mammalian mitoribosome biogenesis. Mitochondrion.

[14] Bassler J., Hurt E. (2019). Eukaryotic Ribosome Assembly. Annu. Rev. Biochem..

[15] Lingaraju M., Schuller J.M., Falk S., Gerlach P., Bonneau F., Basquin J., Benda C., Conti E. (2019). To Process or to Decay: A Mechanistic View of the Nuclear RNA Exosome. Cold Spring Harb. Symp. Quant. Biol..

[16] Lezzerini M., Penzo M., O’Donohue M.F., Marques Dos Santos Vieira C., Saby M., Elfrink H.L., Diets I.J., Hesse A.M., Coute Y., Gastou M. (2020). Ribosomal protein gene RPL9 variants can differentially impair ribosome function and cellular metabolism. Nucleic Acids Res..

[17] Ferretti M.B., Karbstein K. (2019). Does functional specialization of ribosomes really exist?. RNA.

[18] Zhou X., Liao W.J., Liao J.M., Liao P., Lu H. (2015). Ribosomal proteins: Functions beyond the ribosome. J. Mol. Cell Biol..

[19] Thelen N., Defourny J., Lafontaine D.L.J., Thiry M. (2021). Visualization of Chromatin in the Yeast Nucleus and Nucleolus Using Hyperosmotic Shock. Int. J. Mol. Sci..

[20] Paternoga H., Fruh A., Kunze R., Bradatsch B., Bassler J., Hurt E. (2020). Mutational Analysis of the Nsa2 N-Terminus Reveals Its Essential Role in Ribosomal 60S Subunit Assembly. Int. J. Mol. Sci..

[21] Maksimova E.M., Korepanov A.P., Kravchenko O.V., Baymukhametov T.N., Myasnikov A.G., Vassilenko K.S., Afonina Z.A., Stolboushkina E.A. (2021). RbfA Is Involved in Two Important Stages of 30S Subunit Assembly: Formation of the Central Pseudoknot and Docking of Helix 44 to the Decoding Center. Int. J. Mol. Sci..

[22] Goldfarb K.C., Cech T.R. (2017). Targeted CRISPR disruption reveals a role for RNase MRP RNA in human preribosomal RNA processing. Genes Dev..

[23] Lindahl L., Bommankanti A., Li X., Hayden L., Jones A., Khan M., Oni T., Zengel J.M. (2009). RNase MRP is required for entry of 35S precursor rRNA into the canonical processing pathway. RNA.

[24] Schmitt M.E., Clayton D.A. (1993). Nuclear RNase MRP is required for correct processing of pre-5.8S rRNA in Saccharomyces cerevisiae. Mol. Cell. Biol..

[25] Chu S., Archer R.H., Zengel J.M., Lindahl L. (1994). The RNA of RNase MRP is required for normal processing of ribosomal RNA. Proc. Natl. Acad. Sci. USA.

[26] Robertson N., Shchepachev V., Wright D., Turowski T.W., Spanos C., Helwak A., Zamoyska R., Tollervey D. (2022). A disease-linked lncRNA mutation in RNase MRP inhibits ribosome synthesis. Nat. Commun..

[27] Lygerou Z., Allmang C., Tollervey D., Seraphin B. (1996). Accurate processing of a eukaryotic precursor ribosomal RNA by ribonuclease MRP in vitro. Science.

[28] Li X., Zengel J.M., Lindahl L. (2021). A Novel Model for the RNase MRP-Induced Switch between the Formation of Different Forms of 5.8S rRNA. Int. J. Mol. Sci..

[29] Kofler L., Prattes M., Bergler H. (2020). From Snapshots to Flipbook-Resolving the Dynamics of Ribosome Biogenesis with Chemical Probes. Int. J. Mol. Sci..

[30] Kellogg M.K., Miller S.C., Tikhonova E.B., Karamyshev A.L. (2021). SRPassing Co-translational Targeting: The Role of the Signal Recognition Particle in Protein Targeting and mRNA Protection. Int. J. Mol. Sci..

[31] Martin-Villanueva S., Gutierrez G., Kressler D., de la Cruz J. (2021). Ubiquitin and Ubiquitin-Like Proteins and Domains in Ribosome Production and Function: Chance or Necessity?. Int. J. Mol. Sci..

[32] Pecoraro A., Pagano M., Russo G., Russo A. (2021). Ribosome Biogenesis and Cancer: Overview on Ribosomal Proteins. Int. J. Mol. Sci..

[33] Ulirsch J.C., Verboon J.M., Kazerounian S., Guo M.H., Yuan D., Ludwig L.S., Handsaker R.E., Abdulhay N.J., Fiorini C., Genovese G. (2018). The Genetic Landscape of Diamond-Blackfan Anemia. Am. J. Hum. Genet..

[34] Kang J., Brajanovski N., Chan K.T., Xuan J., Pearson R.B., Sanij E. (2021). Ribosomal proteins and human diseases: Molecular mechanisms and targeted therapy. Signal Transduct. Target. Ther..

[35] DeLeo K.R., Baral S.S., Houser A., James A., Sewell P., Pandey S., DiMario P.J. (2021). Drosophila to Explore Nucleolar Stress. Int. J. Mol. Sci..

[36] Martinez-Seidel F., Beine-Golovchuk O., Hsieh Y.C., Eshraky K.E., Gorka M., Cheong B.E., Jimenez-Posada E.V., Walther D., Skirycz A., Roessner U. (2021). Spatially Enriched Paralog Rearrangements Argue Functionally Diverse Ribosomes Arise during Cold Acclimation in Arabidopsis. Int. J. Mol. Sci..

